# Improved Binary Grasshopper Optimization Algorithm for Feature Selection Problem

**DOI:** 10.3390/e24060777

**Published:** 2022-05-31

**Authors:** Gui-Ling Wang, Shu-Chuan Chu, Ai-Qing Tian, Tao Liu, Jeng-Shyang Pan

**Affiliations:** 1 College of Computer Science and Engineering, Shandong University of Science and Technology, Qingdao 266590, China; a70g26@163.com (G.-L.W.); scchu0803@gmail.com (S.-C.C.); stones12138@163.com (A.-Q.T.); taoliu0201@sdust.edu.cn (T.L.); 2 College of Science and Engineering, Flinders University, Adelaide 5042, Australia; 3 Department of Information Management, Chaoyang University of Technology, Taichung 413, China

**Keywords:** grasshopper optimization, binary version, transfer function, feature selection

## Abstract

The migration and predation of grasshoppers inspire the grasshopper optimization algorithm (GOA). It can be applied to practical problems. The binary grasshopper optimization algorithm (BGOA) is used for binary problems. To improve the algorithm’s exploration capability and the solution’s quality, this paper modifies the step size in BGOA. The step size is expanded and three new transfer functions are proposed based on the improvement. To demonstrate the availability of the algorithm, a comparative experiment with BGOA, particle swarm optimization (PSO), and binary gray wolf optimizer (BGWO) is conducted. The improved algorithm is tested on 23 benchmark test functions. Wilcoxon rank-sum and Friedman tests are used to verify the algorithm’s validity. The results indicate that the optimized algorithm is significantly more excellent than others in most functions. In the aspect of the application, this paper selects 23 datasets of UCI for feature selection implementation. The improved algorithm yields higher accuracy and fewer features.

## 1. Introduction

Recent years have witnessed a spurt of the development of informatics, and the data scale of applications such as statistical analysis and data mining is becoming larger and larger. Accordingly, the number of features obtained from the dataset is also increasing. However, some features may be irrelevant or redundant, independent of the final classification goal [1]. Therefore, it is necessary to reduce the dimension of the data and obtain representative features before the classification task. Data preprocessing can smooth out noisy and incomplete data, detect redundancy, and have strong robustness. As an essential preprocessing function, feature selection can clean and remove useless data features effectively [2]. Thus, FS plays an essential role in dimensionality reduction and improving classification performance.

FS is an effective strategy to reduce dimensionality and eliminate noisy and unreliable data [3]. It refers to finding feature-related subsets from a large set of attributes. There are $2^{N}-1$ possible feature subsets in a dataset with *N* features. Davies proves that the search for the smallest subset of features is an NP problem, which means there is no guarantee of finding an optimal solution other than an exhaustive search [4,5]. However, when the number of features is large, an exhaustive search cannot be applied in practical applications because of the large amount of calculation. Therefore, people are committed to using a heuristic search algorithm to find the suboptimal solution. Many studies have attempted to model feature selection as a combinatorial optimization problem. The objective function can be classification accuracy or some other criterion that considers the best trade-off between the number of extracted features and efficiency [6].

The meta-heuristic algorithms are used to find the optimal or satisfactory solution to complex optimization problems [7,8,9]. The principles of optimization algorithms are revealed through knowledge of relevant behaviors and experiences in biological, physical, and other system domains. In 1991, an Italian scholar proposed the theory of ant colony optimization (ACO) [10]. Since then, swarm intelligence has been formally proposed as a theory. Swarm intelligence takes advantage of group information. It has been extensively used in optimization problems. In 1995, some scholars presented the particle swarm optimization (PSO) algorithm [11], and then the research on this subject was carried out rapidly. The cat swarm optimization based on feline predation strategies was introduced in 2006 [12]. In 2010, fish migration optimization (FMO) emerged, which integrated migration and swim models into the optimization process [13]. In 2017, Saremi et al. proposed the grasshopper optimization algorithm (GOA) [14]. GOA solves optimization problems by mathematically modeling and simulating the behavior of grasshopper swarms in nature. Compared with other existing algorithms, GOA has a higher search efficiency and faster convergence speed. It also solves the continuous problem of finding the best shape for a 52-bar truss and a 3-bar truss. Over recent years, more sophisticated algorithms have been put forward, such as the sparrow search algorithm (SSA) [15], seagull optimization algorithm (SOA) [16], quasi-affine transformation evolution with external archive (QUATRE-EAR) [17], and polar bear optimization algorithm (PBO) [18].

Nonetheless, many optimization problems are discrete problems, such as FS. Conventional methods can not satisfy practical needs, so binary algorithms are needed to solve this problem. Up till now, scholars have proposed many binary algorithms and achieved quite fruitful results. Among them, the well-known PSO algorithm and its binary variants have been put into feature selection [19,20,21,22]. A binary whale optimization algorithm was presented to handle discrete problems in this work [23]. Binary fish migration optimization algorithm (ABFMO) [24] and improved binary symbiotic organism search algorithm (IBSOS) using transfer function also solved the FS problem [25]. Accordingly, the pigeon flock optimization algorithm (PIO) and the gray wolf optimization algorithm (GWO) were improved for better application in feature selection [26,27,28]. The pigeon flock optimization algorithm simulates the pigeons’ homing behavior. Based on the binary pigeon flock optimization algorithm (BPOI), Tian et al. proposed improved binary pigeon-inspired optimization (IBPIO) [29]. They offered a new speed update equation and finally achieved excellent results. Additionally, the binary approach enabled the GWO to be applied to discrete problems [30,31]. The novel gray wolf optimization algorithm (BGWO) added a new parameter update equation to enhance the search capability [32]. Besides, the author gave five transfer functions for the feature selection of UCI datasets. Beyond that, the binary version of GOA was also used to solve the FS problem [33]. Hichem et al. proposed a Novel Binary GOA (NBGOA) by modeling position vectors as binary vectors in [34]. Pinto et al. [35] developed a binary GOA based on the percentile concept for solving the Multidimensional Knapsack Problem (MKP). Moreover, BGOA-M, a binary GOA algorithm based on the mutation operator, was introduced for the FS problem [36].

The sigmoid transfer function is a common method used when converting algorithms to binary versions [37,38]. Some scholars suggested improved binary EO (BEO) for FS problems using the sigmoid function [39]. The authors presented binary MPA (BMPA) and its improved versions using sigmoid and eight transfer functions [6]. In the binary grasshopper optimization algorithm (BGOA), the authors used the sigmoid transfer function to convert space to binary [36]. It has been well applied in feature selection. However, there is a weakness in the original BGOA. The conversion probability of position only accounts for a small range, which can not satisfy the exploration requirement of the algorithm. Thus, this paper presents an improved BGOA to avoid this situation. In the first place, the improved BGOA optimizes the step size in the original BGOA. Secondly, two sigmoid-based and one V-shaped transfer function are proposed based on the new step size. To evaluate the effectiveness of the improved algorithm, 23 well-known datasets are used for experiments. For the performance analysis, the improved BGOA is compared with BGOA, BPSO, and BGWO. Experiments prove that the proposed algorithm performs excellently than the original BGOA in the FS problem. There are the main contributions:

1. The range of step size variables in the original BGOA is optimized.
2. Three new transfer functions and two position conversion formulas are proposed based on the new step size.
3. The efficiency of the improved algorithm is examined by several experiments on 23 benchmark functions [40].
4. The improved algorithm achieves satisfactory results in feature selection application.

The rest of this paper is shown below. Section 2 is the preliminaries, which contain GOA and the original BGOA. Section 3 presents the improved version of BGOA. Section 4 shows the effect of the improved BGOA on 23 benchmark functions. Section 5 describes the application of the improved BGOA to feature selection. Section 6 analyzes the results of feature selection. Section 7 gives a discussion of this paper.

## 2. Preliminaries

GOA has been maturely applied to continuity problems. Its binary variants have also been gradually refined. This section introduces the standard GOA and the BGOA based on the sigmoid transfer function.

### 2.1. GOA

Grasshoppers are incompletely metamorphosed insects, consisting of three stages: egg, worm, and adult. Grasshoppers are a worldwide agricultural pest and generally occur individually. Nevertheless, they are swarming organisms that excel in periodic population outbreaks and can migrate over long distances. Grasshoppers are usually found in the worm and adult stages. The adult grasshoppers have solid hind feet, which causes tremendous damage to agriculture, forestry, and animal husbandry. They are adept at jumping and flying through the air with an extensive wide range of movement. In addition to migration, grasshoppers are also characterized by their predation process. Nature-inspired optimization algorithms have two phases: exploration and exploitation. Exploration is a large-scale search to prevent falling into a local optimum, while the exploitation phase is a small-scale search to find the optimal solution [41,42]. Grasshoppers can instinctively perform these two steps to find the target. Furthermore, according to the grasshopper’s characteristics, GOA has a unique adaptive mechanism. It can effectively regulate the global and partial search process with higher search accuracy. This phenomenon is mathematically modeled by Saremi et al. [43]:

(1) $$X_{i}=S_{i}+G_{i}+A_{i},$$

here $X_{i}$ represents the position of the *i*-th grasshopper at this time. $S_{i}$ represents the influence factor of two individuals, $G_{i}$ is the gravitational influence. $A_{i}$ is the wind influence. Each operator is multiplied by a random number from 0 to 1 to enhance the randomness, as shown in Equation (2):

(2) $$X_{i}=k_{1}S_{i}+k_{2}G_{i}+k_{3}A_{i}.$$

The details of $S_{i}$ for the social interaction operator are as below:

(3) $$S_{i}=\sum_{\begin{array}{c} j=1\\ j\neq i \end{array}}^{N}s\left(d_{ij}\right)\hat{d_{ij}},$$

where $d_{ij}$ is the distance between the *i*-th and *j*-th grasshoppers, function s calculates the intensity of social interaction, $\hat{d_{ij}}$ =$\frac{x_{j}-x_{i}}{d_{ij}}$ is the unit vector between the *i*-th and *j*-th grasshoppers. Where $x_{i}$ and $x_{j}$ represent the positions of the *i*-th and *j*-th grasshoppers, respectively. The *s* function is defined as shown in Equation (4):

(4) $$s\left(a\right)=fe^{\frac{-a}{l}}-e^{-a},$$

where *f* is the attraction strength, *l* is the attraction length scope. The value of *s* is negative to indicate mutual repulsion, while positive indicates mutual attraction between grasshoppers. 0 means that they are in their comfort zone. The value of *f* is 0.5, and the value of *l* is 1.5. When two grasshoppers are too far apart, the force does not exist. Therefore, the distance has to be normalized. In his paper, the author does not take gravity into account. The wind direction is toward the best value. The final position formula is shown in Equation (5). The Pseudocode of GOA is given in Algorithm 1.

(5) $$X_{i}^{d}=c\left(\sum_{\begin{array}{c} j=1\\ j\neq i \end{array}}^{N}c\frac{ub_{d}-lb_{d}}{2}s\left(\left|X_{j}^{d}-X_{i}^{d}\right|\right)\frac{x_{j}-x_{i}}{d_{ij}}\right)+T_{d}.$$

**Algorithm 1:** Pseudocode of GOA
1:Initialize $C_{max}$, $C_{min}$ (two extreme values of parameter *c*), Max_iter (iterations’ maximum) and *N* (population of grasshoppers)2:Initialize the position of each grasshopper: $X_{i}$ (*i* = 1,2, …, *n*)3:Set the best solution as Target4:**while***t* ≤ Maxiter **do**5:    Update *c* with Equation (6)6:    **for** each agent **do**7:        Normalize the distance among two individuals to [1, 4]8:        Update $X_{i}$ using Equation (5)9:        Update Target, if a better value is obtained10:    **end for**11:    t=t+112:
**end while**
13:Output Target


The $ub_{d}$ and $lb_{d}$ are the boundary values of the *d*-th dimension, respectively. $T_{d}$ is the optimal value found so far, and *N* represents the number of populations. An explanation of the calculation of *c* in Equation (5) can be found below:

(6) $$c=C_{max}-t\left(\frac{C_{max}-C_{min}}{T}\right),$$

where $C_{max}$ is the maximum and $C_{min}$ is the minimum. The *t* is a number of the current iterations. *T* is the total iterations. It should be easy to see that *c* becomes smaller as the number of iterations increases. The first *c* can narrow the search area around the target with the increased number of iterations. The second *c* is used to reduce the gravitational force or repulsion between grasshoppers. In the text, $C_{max}=1$, $C_{min}=0.00001$.

From Equation (5), we can know that the new position of the *i*-th grasshopper is not only related to its current position but also related to the current situation of all other grasshoppers and the interaction forces between individuals. The adaptive mechanism of the algorithm can balance the global and local search. It has an excellent optimization-seeking ability.

### 2.2. BGOA

The search space of GOA is continuous. Thus the position can be moved randomly. However, in binary space, the position can only take 0 or 1. Mafarja et al. used the sigmoid transfer function in the paper to implement the binary conversion:

(7) $$T\left(\Delta X_{t}\right)=\frac{1}{1+e^{-\Delta X_{t}}},$$

here $\Delta X_{t}$ is the first part of Equation (5), similar to the velocity variable in the PSO algorithm, which is called step size. The absolute value of $\Delta X_{t}$ can be considered as the distance between the updated position of the grasshopper and the target position in the *d*-th dimension. A conversion probability is obtained based on the transfer function. Accordingly, the formula for updating the grasshopper’s position is also changed through Equation (7) and Equation (8):

(8) $$X_{t+1}^{d}=\left\{\begin{array}{cc} 1 & if\;r_{1}<T\left(\Delta X_{t+1}^{d}\right)\\ 0 & if\;r_{1}\geq T\left(\Delta X_{t+1}^{d}\right) \end{array}\right.,$$

where $r_{1}$ belongs to [0, 1], $X_{t+1}^{d}$ is the position of *d-th* dimensional after the *t*-th iteration.

## 3. Analysis and the Improvement of Binary Grasshopper Optimization Algorithm

The standard GOA algorithm and its modified versions have worked and achieved good results on continuous problems. Feature selection can be seen as a binary problem of selecting the appropriate 0/1 string. The initial length of the string is the whole amount of features in the original dataset. 0 represents the unselected attribute, 1 for a selected attribute. Additionally, The transfer function is a common and classical method in converting continuous to binary space [44].

**Figure 1:**
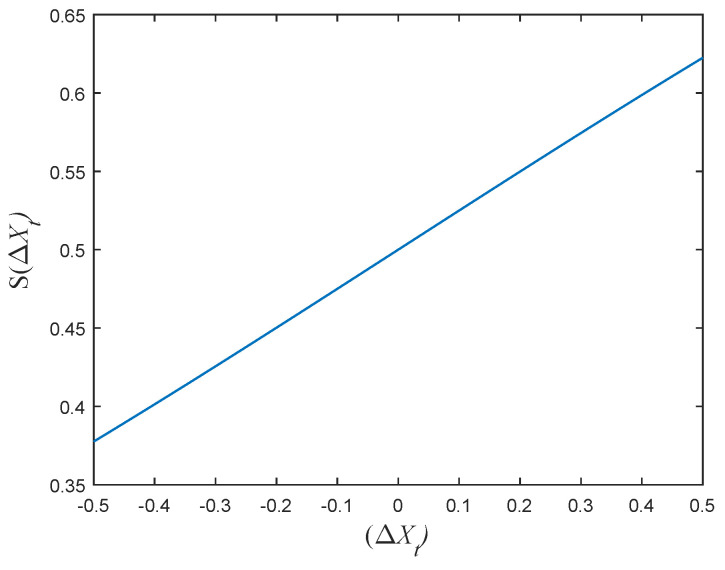
Curve for the range of values.

The original BGOA used step size and transfer function for binary conversion to obtain specific results. From the analysis in Section 2, we know that parameter *c* and the step size become smaller as the number of iterations increases. After debugging the code and preserving the decimal places, the range of step size is found in [−0.3, 0.4], which indicates that the conversion probability is always taken to be a small part of [0, 1]. The curve is shown in Figure 1. Beyond that, the parameter $r_{1}$ in Equation (8) is a random number; thus, it may not be conducive to the position update in the former exploration stage. The ideal result is that the individuals in the population can randomly transform their positions in the binary pattern. To avoid the situation, this paper improves the performance of BGOA by modifying the transfer function. This manuscript proposes a new step size variable and three improved transfer functions. The first two transfer functions are based on the sigmoid transfer function, and the third is a V-shaped function.

The step size is modified to consider both the range of population positions and the uniformity of particles falling around 0.5 to ensure fairness. When the step size takes a value close to 6, the conversion probability is nearly 1. Therefore, increase the step size ΔX to 20 times, and change the range to [−6, 6]. The new transfer functions are proposed based on the new range. These transfer functions have two extremes close to 0 and 1 on [−6, 6], which has strong randomness in converting the binary position. The range on both sides of the 0 point is also evenly distributed. Here we set *B* to 20.

When the grasshopper updates its position, the transition probability is obtained according to Equation (9), which we refer to as BGOAS1:

(9) $$S1\left(B\Delta X_{t}\right)=\frac{1}{1+e^{-\frac{17}{15}\left(1+B\Delta X_{t}\right)}}.$$

Equation (10) is called BGOAS2:

(10) $$S2\left(B\Delta X_{t}\right)=\frac{\sqrt{15}}{4}\frac{1}{1+e^{-\frac{\Pi }{3}\left(B\Delta X_{t}\right)}},$$

and Equation (11) is called BGOAV:

(11) $$V\left(B\Delta X_{t}\right)=2\sqrt{\frac{2}{3}}\frac{\left|\tanh\left(B\Delta X_{t}\right)\right|}{\tanh4}.$$

The new position is derived according to Equation (12) or Equation (13):

(12) $$X_{t+1}^{d}=\left\{\begin{array}{cc} 1 & if\;r<S1\left(B\Delta X_{t}\right)\vee r<S2\left(B\Delta X_{t}\right)\\ 0 & if\;r\geq S1\left(B\Delta X_{t}\right)\vee r\geq S2\left(B\Delta X_{t}\right) \end{array}\right.$$

(13) $$X_{t+1}^{d}=\left\{\begin{array}{cc} {\left(X_{t}^{d}\right)}^{-1} & if\;r<V(B\Delta X_{t})\\ X_{t}^{d} & if\;r\geq V(B\Delta X_{t}) \end{array}\right..$$

**Figure 2:**
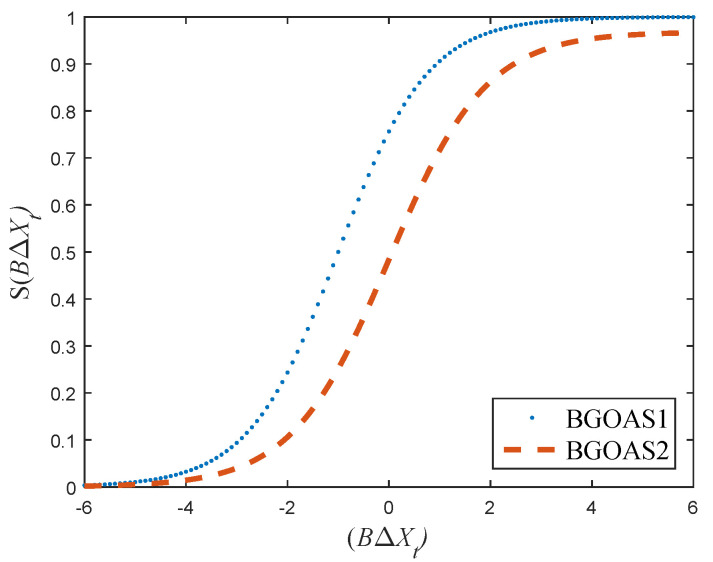
BGOAS1 and BGOAS2 tranafer functions.

**Figure 3:**
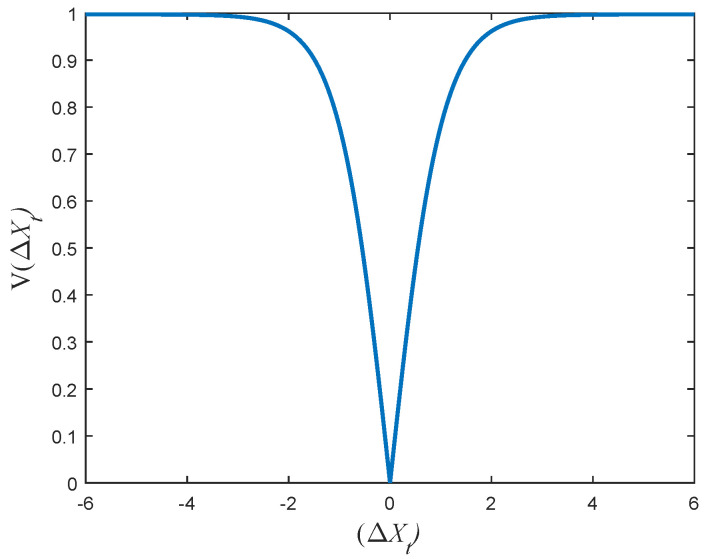
BGOAV transfer function.

From the above description, we can learn that the new position of the grasshoppers depends on the current position of all grasshoppers. It is finally derived from the position conversion probability. Compared with the existing BGOA, the proposed methods in this paper have better exploration ability and randomness. New transfer functions are shown in Figure 2 and Figure 3. The Pseudocode of the new BGOA is displayed in Algorithm 2.
**Algorithm 2:** Pseudocode of new BGOA1:Initialize $C_{max}$, $C_{min}$ (two extreme values of parameter *c*), Max_iter (iterations’ maximum) and *N* (population of grasshoppers)2:Set the best solution as Target3:**while***t* ≤ Maxiter **do**4:    Update *c* with Equation (6)5:    **for** each agent **do**6:        Normalize the distance among two individuals to [1, 4]7:        Calculate probability using Equation (9) or Equation (10) or Equation (11)8:        Update $X_{i}$ using Equation (12) or Equation (13)9:        Update Target, if a better value is obtained10:    **end for**11:    t=t+112:**end while**13:Output Target

## 4. Experimental Results

The validity of the new algorithm is verified in this section. There are many excellent test functions like benchmarks in the BBOB workshop, which support algorithm developers and practitioners alike by automating benchmarking experiments for black-box optimization algorithms [45,46,47]. This manuscript uses 23 benchmark test functions to demonstrate the effectiveness of the proposed algorithm. Among them, f1–f7 are unimodal benchmark test functions, f8–f13 are multimodal benchmark test functions, and f14–f19 are fixed-dimension benchmark test functions. The details of each test function are presented in Table 1, Table 2 and Table 3. Space means the search space of the population, Dim is the function’s dimension, and TM is their theoretical optimum. The settings of all parameters required by the algorithms are in Table 4.

The improved algorithm is compared with BGOA, BPSO, and BGWO. The mean and standard deviation (std) of the test functions are given in Table 5 and Table 6. If the improved algorithm works better than or the same as the original one, then we put the good result in bold font. For example, for f12, the values obtained using BGOAS1, BGOAS2 and BGOAV are smaller than BGOA, so the first three values are indicated in bold font.

As can be seen, the improved algorithm has an obvious advantage over BGOA, BPSO, and BGWO in the mean values of fitness results obtained on the first 13 test functions. The result indicates that the improved algorithm is more effective in solving high-dimensional problems. On the fixed-dimension functions, values obtained by the six algorithms are almost the same. It illustrates that the improved strategy is not the most efficient for addressing the low-dimensional problem.

For the unimodal test functions, there is only one optimal solution. Consequently, they can effectively check the convergence rate of the algorithms. Table 5 and Table 6 show that the results of the proposed algorithm BGOAV outperform the compared algorithms in all seven unimodal functions. The mean and std are the smallest. In f2, f3, and f6, BGOAS1 and BGOAS2 also obtain the optima of considered algorithms.

Functions f8-f13 are multimodal test functions. These functions have many local optima and are suitable for testing the ability of the algorithm to avoid local optima. BGOAS1, BGOAS2, and BGOAV perform well in these functions. BGOAV outperforms the other algorithms in both the mean and standard deviation of the results. Functions f9 and f11 reach the theoretical optimum with BGOAS1, BGOAS2, and BGOAV. As to f8 and f12, the proposed methods are closer to the optimum than BPSO and BGWO. Moreover, for f13, the best result is obtained using BGOAV, BPSO, and BGWO. In other words, the proposed strategy to improve the step size produces good results and prevents the algorithms from falling into local optima.

Functions f14–f23 are the fixed-dimension functions. It is evident from the results that the mean and standard deviation obtained by all algorithms are almost the same. Only f20 does the BGOA get a value closer to the theoretical one. It proves that on the fixed-dimension functions, the new algorithm has no special advantage over BGOA, BPSO, and BGWO. It is due to the low-dimensional and simple structure of the function, while the improved strategies are better at high-dimensional and complex problems.

To judge whether the results of the improved strategies differ from the best results of the other algorithms, Wilcoxon rank-sum test and Friedman test were performed at a 5% significance level in this experiment. We assume that there are no significant differences between the algorithms. If the p-value is smaller than 0.05, the hypothesis is not valid, and the original hypothesis is overturned. It can be identified from Table 7 that for f1–f4, the p-values obtained by BGOAS1, BGOAV, BGWO, and BPSO are smaller than 0.05, which means that there is a significant difference between BGOAS1, BGOAV, and BGOA. Data in Table 8 show that the p-value is not greater than 0.05 in f1–f3 and f5–f13, which could be considered strong evidence against the null hypothesis. The data suggests that there is a significant difference between these algorithms. This result illustrates new algorithm is superior to BGOA, BPSO, and BGWO in these 12 functions. It can be argued that the improved methods outperform the compared algorithms overall.

It is easy to see that the improved strategies promote the exploration and exploitation of BGOA. Moreover, it heightens the competitiveness of the algorithm in finding optimal solutions to functions. In the next section, this paper applies the improved algorithm to a real problem to study its practicability in FS.

## 5. Application of Feature Selection

Feature selection is a major function in the pre-processing part of data mining. It can remove irrelevant and redundant data from the dataset [48]. Researchers usually focus on the method with high precision and low features. In this section, the improved strategies (BGOAS1, BGOAS2, BGOAV) are exploited in feature selection for classification problems. It can be found that the improved strategies obtain better results and yield more accurate subsets of features.

Twenty-three datasets are selected for feature selection from UCI machine learning [49], each with different attributes and instance data. In addition, this paper uses a wrapper-based method for feature selection. The detailed information of the 23 datasets is introduced in Table 9.

K-Nearest Neighbor (KNN) classification algorithm is the most commonly used classification algorithm in data mining [50]. KNN is a supervised learning method with a simple mechanism: given a testing sample, find the K nearest training samples based on some distance metric, and then use these K “neighbors” to make predictions. Typically, voting can be used to classify the test samples with the most frequent of the K neighbors into one class. The distance metric between different samples generally selects Euclidean distance or Manhattan distance [51]. The computational method is shown in Equation (14):

(14) $$L_{p}\left(x,y\right)={\left(\sum_{\begin{array}{c} i=1 \end{array}}^{n}{\left|x_{i}-y_{i}\right|}^{p}\right)}^{\frac{1}{p}},$$

where *p* is a variable constant. When p=1, *L* represents the Manhattan distance, and if p=2, *L* refers to the Euclidean distance. The $x_{i}$ and $y_{i}$ represent two different instances in the set, respectively.

The basic idea of cross-validation is to split the original data into training and testing sets [52]. The former is used for training the model, and the testing set is used for model validation. K-fold cross-validation divides all initial samples into K equally sized subsets. Then traverse the K subsets in turn. Each time the current subset is used as the verification set, and all other samples as the training set to train and evaluate the model. Finally, the average value of K evaluations is taken as the final evaluation criterion of the model. 20 is the maximum value of K. Generally, 10 is sufficient [53].

The error rate and accuracy of classification are crucial evaluation indicators in classification prediction. This paper uses Equation (15) as the fitness function:

(15) $$Fitness=\mu *errate_{\left(KNN\right)}+\left(1-\mu \right)*\left(\frac{SeF}{AlF}\right),$$

where $errate_{\left(KNN\right)}$ denotes the classification error rate after K-fold cross-validation, which is explained in Equation (16). Parameter μ is often taken as 0.99. SeF is the subset feature after feature selection, AlF is the number of features for the dataset:

(16) $$errate_{\left(KNN\right)}=\frac{Enum}{Enum+Cornum}.$$

*Enum* and *Cornum* are the error and the correct number of the classification, respectively.

It can be seen from Equation (15) that the fitness function aims to find the combination of features with maximum classification performance and a minimum number of selected features. It is converted into a minimization problem by using the error rate instead of the classification accuracy and using the selected feature ratio instead of the unselected feature ratio.

Wrapper-based method for feature selection directly uses the performance of the final model as the evaluation criterion for the feature subsets. In this paper, the KNN is used as a classification to ensure the goodness of the selected features. The improved BGOAS1, BGOAS2, and BGOAV are used as search methods that can adaptively search the feature space to achieve higher feature evaluation criterion. A single dimension in the search space represents a feature, so the grasshopper’s position represents a combination of features or a solution. It is noted that the higher feature evaluation criterion is expressed as smaller fitness values in Equation (15).

## 6. Results of Feature Selection

The improved algorithm, BGOA, BPSO, and BGWO algorithms are applied to feature selection. All the population sizes are set to 30. The iterations are 100 and run 15 times on each dataset. The value of *K* in KNN is taken as 10. Table 10 shows the feature selection fitness values. Table 11 records the number of feature selections. Table 12 describes the accuracy of the feature selection. Wilcoxon rank-sum test and Friedman test are examined for the mean accuracy and fitness values in Table 13 and Table 14.

Table 10 shows that the new strategies have great advantages. The improved strategies obtain better results than the original BGOA on 15 datasets. In Air, Astura, Breast, and Segmentation datasets, new strategies outperform BPSO. Only in 5 datasets does the original BGOA obtain a better value. In Appendicitis, Breast, Bupa, Diabetes, and Glass datasets, the original BGOA gets the best result. The number of selected feature subsets presented in Table 11 also supports the claim that the improved algorithm has better performance than compared algorithms. It is worth mentioning that BGOAS1 performs well in the number of selections, with the smallest subset of features selected in 14 datasets. On all 23 datasets, the improved algorithm obtained better or equal results than BGOA, BPSO, and BGWO. The accuracy of feature selection is shown in Table 12. BGOAS1 achieves exceptionally high accuracy on the 8 datasets: Balancescale, Bupa, Cloud, Diabetes, and Heartstatlog datasets. Compared with the original algorithm, the accuracy of feature selection is improved by about 3%. Accordingly, BGOAS2 and BGOAV obtain higher accuracy than BGOA on 10 and 6 datasets. Among them, the accuracy of the Vowel dataset reaches 1. On the Air, Appendicitis, Breast, WDBC, and Zoo datasets, the performance of the six algorithms is comparable.

Table 13 lists the Wilcoxon rank-sum test of the new strategies with the original BGOA. Values in Air, Cleve, Segmentation, Thyroid, and Ecoli datasets are smaller than 5%. From the Friedman test in Table 14, it can be obtained that values in WDBC, Bupa, Segmentation, Jain, Vowel, and Sonar datasets are smaller than 0.05. Therefore, it can be considered that there are significant differences between these algorithms. The results in these tables prove the better validity and feasibility of the improved algorithm.

## 7. Discussion

The binary grasshopper optimization algorithm solves discrete problems such as feature selection. This paper presented three improved versions of the binary grasshopper optimization algorithm for feature selection. A new step size variable and three transfer functions were introduced to optimize the algorithm’s exploration capability in binary space. Besides, this paper has done several tests on 23 benchmark test functions to certify the algorithm’s feasibility. The improved algorithm shows preferable performance in high-dimensional functions. Subsequently, simulation experiments for feature selection are conducted. In the 23 UCI datasets, the KNN and 10-fold cross-validation are adopted to address the wrapper-based feature selection problem. The improved algorithms are more competitive than the original BGOA, BPSO, and BGWO regarding fitness values and selected subsets.

It should be noted that the method of this paper has applied only to feature selection. Thus, it can address other binary combinatorial optimization problems, including task scheduling and traveling salesman problems. Apart from that, many excellent benchmarks in the BBOB workshop may be very effective for the further improvement of BGOA. Hence, more in-depth studies like using benchmarks in the BBOB workshop to examine the algorithm will be conducted in the future. Finally, the improved algorithm does not perform well on low-dimensional functions, and the binary conversion increases the computing time. Future work involving shortening the running time of the algorithm and improving its ability to solve low-dimensional problems is expected to execute.

## Figures and Tables

**Table 1:** Unimodal test functions.

Num	Name	Space	Dim	TM
1	Sphere	[−100, 100]	30	0
2	Schwefel’s function 2.21	[−10, 10]	30	0
3	Schwefel’s function 1.2	[−100, 100]	30	0
4	Schwefel’s function 2.221	[−100, 100]	30	0
5	Rosenbrock	[−30, 30]	30	0
6	Step	[−100, 100]	30	0
7	Dejong’s noisy	[−1.28, 1.28]	30	0

**Table 2:** Multimodal test functions.

Num	Name	Space	Dim	TM
8	Schwefel	[−500, 500]	30	−12,569
9	Rastringin	[−5.12, 5.12]	30	0
10	Ackley	[−32, 32]	30	0
11	Griewank	[−600, 600]	30	0
12	Generalized penalized 1	[−50, 50]	30	0
13	Generalized penalized 2	[−50, 50]	30	0

**Table 3:** Fixed-dimension test functions.

Num	Name	Space	Dim	TM
14	Fifth of Dejong	[−65, 65]	2	1
15	Kowalik	[−5, 5]	4	0.00030
16	Six-hump camel back	[−5, 5]	6	−1.0316
17	Branins	[−5, 5]	2	0.398
18	Goldstein–Price	[−2, 2]	2	3
19	Hartman 1	[0, 10]	3	−3.86
20	Hartman 2	[0, 1]	6	−3.32
21	Shekel 1	[0, 1]	4	−10.1532
22	Shekel 2	[0, 1]	4	−10.4028
23	Shekel 3	[0, 1]	4	−10.5363

**Table 4:** Parameters and values.

Parameter	Value
Cmax	1
Cmin	0.00004
C1	2
C2	2
wmax	0.9
wmin	0.2
SigmaMax	1
SigamaMin	0.1
Vmax	6
Vmin	−6
popnum	30
Max_iter	500

**Table 5:** The result of mean values.

Functions	BGOA_S1	BGOA_S2	BGOA_V	BGOA	BPSO	BGWO
f1	10.0000	**9.0000**	**7.8000**	10.0000	4.6000	3.2000
f2	**0.0000**	0.8000	**0.0000**	0.6000	0.0000	0.0000
f3	0.4000	**0.0000**	**0.0000**	0.2000	0.0000	0.0000
f4	0.2000	0.6000	**0.0000**	0.2000	0.0000	0.2000
f5	20.8000	**2.4000**	**0.0000**	20.0000	0.0000	0.0000
f6	2.8500	**1.2500**	**1.2500**	2.4500	1.2500	1.2500
f7	**0.0062**	0.2078	**0.0016**	1.0064	0.0055	0.0002
f8	**−4.2074**	**−4.2074**	**−4.2074**	−4.2074	−3.8708	−3.8708
f9	0.8000	0.6000	**0.0000**	0.4000	0.0000	0.2000
f10	1.0267	1.0267	$8.88\times 10^{-16}$	0.3422	$8.88\times 10^{-16}$	$8.88\times 10^{-16}$
f11	0.0861	**0.0197**	**0.0000**	0.0394	0.0000	0.0000
f12	**4.1233**	**4.1862**	**4.1233**	4.4846	4.1233	4.1233
f13	0.0400	0.0400	$1.35\times 10^{-32}$	0.0600	1.35×10−32	1.35×10−32
f14	**12.6705**	**12.6705**	**12.6705**	12.6705	12.6705	12.6705
f15	**0.1484**	**0.1484**	**0.1484**	0.1484	0.1484	0.1484
f16	**0.0000**	**0.0000**	**0.0000**	0.0000	0.0000	0.0000
f17	**27.7029**	**27.7029**	**27.7029**	27.7029	27.7029	27.7029
f18	**600.0000**	**600.0000**	**600.0000**	600.0000	600.0000	600.0000
f19	−0.3348	−0.3348	−0.3348	−0.3348	−0.3348	−0.3348
f20	−0.1343	−0.1196	**−0.1657**	−0.0989	−0.1657	−0.1469
f21	−4.2205	**−5.0552**	**−5.0552**	−5.0552	−5.0552	−5.0552
f22	−3.4172	**−5.0877**	**−5.0877**	−5.0877	−5.0877	−5.0877
f23	**−5.1285**	**−5.1285**	**−5.1285**	−5.1285	−5.1285	−5.1285

If the improved algorithm works better than or the same as the original BGOA, then we put the good result in bold font.

**Table 6:** The result of std values.

Functions	BGOA_S1	BGOA_S2	BGOA_V	BGOA	BPSO	BGWO
f1	**1.4142**	**1.2247**	**0.8367**	1.4142	0.8944	1.4832
f2	**0.0000**	0.4472	**0.0000**	0.5477	0.0000	0.0000
f3	0.5477	**0.0000**	**0.0000**	0.4472	0.0000	0.0000
f4	0.5477	0.5477	**0.0000**	0.4472	0.0000	0.4472
f5	44.3080	**2.1909**	**0.0000**	44.7214	0.0000	0.0000
f6	0.8944	**0.0000**	**0.0000**	1.0954	0.0000	0.0000
f7	0.0046	0.4485	0.0018	1.4092	0.0053	0.0001
f8	0.4609	0.4609	**0.0000**	0.0000	0.0000	0.0000
f9	**0.4472**	0.5477	**0.0000**	0.5477	0.0000	0.4472
f10	0.9373	0.9373	**0.0000**	0.7653	0.0000	0.0000
f11	0.0887	**0.0441**	**0.0000**	0.0540	0.0000	0.0000
f12	**0.0000**	**0.1405**	**0.0000**	0.4947	0.0000	0.0000
f13	0.0548	0.0548	**0.0000**	0.0548	0.0000	0.0000
f14	0.0000	0.0000	0.0000	0.0000	0.0000	0.0000
f15	0.0000	0.0000	0.0000	0.0000	0.0000	0.0000
f16	0.0000	0.0000	0.0000	0.0000	0.0000	0.0000
f17	0.0000	0.0000	0.0000	0.0000	0.0000	0.0000
f18	0.0000	0.0000	0.0000	0.0000	0.0000	0.0000
f19	0.0000	0.0000	0.0000	0.0000	0.0000	0.0000
f20	**0.0195**	**0.0215**	**0.0000**	0.0272	0.0000	0.0615
f21	1.8663	2.2858	**0.0000**	2.2858	0.0000	0.0000
f22	1.8676	**0.0000**	**0.0000**	2.2877	0.0000	0.0000
f23	0.0000	0.0000	0.0000	0.0000	0.0000	0.0000

If the improved algorithm works better than or the same as the original BGOA, then we put the good result in bold font.

**Table 7:** *p*-value of Wilcoxon rank-sum test.

Functions	BGOA_S1	BGOA_S2	BGOA_V	BPSO	BGWO
f1	0.0556	0.7619	0.0556	0.0079	0.0079
f2	0.5238	0.2063	0.0476	0.0476	0.0476
f3	0.7143	0.2063	0.0476	0.0476	0.0476
f4	0.5238	1	0.1667	0.1667	1
f5	1	1	1	1	1
f6	0.1667	1	1	1	1
f7	0.6905	0.8413	1	0.6905	0.6905
f8	1	1	0.4444	0.4444	0.4444
f9	1	1	0.4444	0.4444	0.4444
f10	0.5238	1	1	1	1
f11	0.0476	0.4444	1	1	1
f12	0.2857	0.5238	0.1667	0.1667	0.1667
f13	1	0.5238	0.4444	0.4444	0.4444
f14	1	1	1	1	1
f15	1	1	1	1	1
f16	1	1	1	1	1
f17	1	1	1	1	1
f18	1	1	1	1	1
f19	1	1	1	1	1
f20	0.3810	0.6825	0.1667	0.1667	1
f21	1	1	1	1	1
f22	1	1	1	1	1
f23	1	1	1	1	1

**Table 8:** The results of Friedman test.

Friedman	Sum of Squares	Degree of Freedom	Mean Squares	*p*-Value
f1	73.2	5	14.62	0.0006
f2	77.3	5	15.46	0.0004
f3	70.7	5	14.14	0.0011
f4	0	5	0	1
f5	80.3	5	16.06	0.0003
f6	72.1	5	14.42	0.0008
f7	73.1	5	14.62	0.0009
f8	69.7	5	13.94	0.0011
f9	78.1	5	15.62	0.0004
f10	72.8	5	14.56	0.0008
f11	71.5	5	14.3	0.0010
f12	73	5	14.6	0.0008
f13	78	5	15.6	0.0004
f14	0	5	0	1
f15	0	5	0	1
f16	0	5	0	1
f17	0	5	0	1
f18	0	5	0	1
f19	0	5	0	1
f20	1.5	5	5	0.4159
f21	6	5	1.2	0.0752
f22	1.5	5	0.3	0.4159
f23	1.5	5	0.3	0.4159

**Table 9:** The details of datasets.

S.no.	Datasets	Instances	Number of Classes (k)	Features of Each Class (d)	Size of Classes
1	Air	359	3	64	107, 103, 149
2	Appendicitis	106	2	7	21, 85
3	Austra	690	2	14	395, 295
4	Balancescale	625	3	4	49, 288, 288
5	Blood	748	2	4	570, 178
6	Breast	277	2	9	196, 81
7	Breast_gy	277	2	9	196, 81
8	Bupa	345	2	6	145, 200
9	Cleve	296	2	13	160, 139
10	Cloud	1024	2	10	627, 403
11	Diabetes	768	8	2	268, 500
12	Ecoli	336	8	8	143, 77, 2, 2, 259, 20, 5, 52
13	Glass	214	6	9	29, 76, 70, 17, 13, 9
14	Heartstatlog	270	2	13	150, 120
15	Jain	373	2	2	276, 97
16	phoneme	5404	2	5	15, 863, 818
17	Robotnavigation	5456	4	25	82, 620, 972, 205, 329
18	Seeds	210	3	7	70, 70, 70
19	segmentation	210	7	18	30, 30, 30, 30, 30, 30, 30
20	Sonar	208	2	60	97, 111
21	Thyroid	215	3	5	150, 35, 30
22	Vowel	871	6	3	72, 89, 172, 151, 207, 180
23	zoo	101	7	16	41, 20, 5, 13, 4, 7, 10

**Table 10:** The result of fitness value.

Dataset	BGOA_S1	BGOA_S2	BGOA_V	BGOA	BPSO	BGWO
Air	0.07380	**0.05781**	0.08481	0.07234	0.08249	0.07068
Appendicitis	0.13522	0.13996	0.14689	0.09244	0.14039	0.13982
Austra	**0.30696**	0.31836	**0.32391**	0.32767	0.32356	0.32189
Balancescale	0.22665	**0.15280**	0.19935	0.17176	0.18391	0.17874
WDBC	**0.04557**	**0.04067**	0.04772	0.04700	0.05628	0.05696
Blood	0.23635	0.23644	0.23634	0.23404	0.22684	0.23165
Breast	0.24452	0.24101	0.25303	0.23613	0.24856	0.24672
Breast_gy	**0.20910**	**0.20922**	**0.22714**	0.23690	0.21706	0.21775
Bupa	0.33957	0.32704	0.36654	0.31300	0.33118	0.32752
Cleve	**0.17600**	0.18140	0.20421	0.19038	0.17440	0.18164
Cloud	**0.00514**	**0.01155**	0.01852	0.01020	0.01450	0.01450
Diabetes	0.24661	0.25597	0.27359	0.24301	0.26049	0.26189
Segmentation	**0.11190**	**0.10644**	0.11683	0.12310	0.11451	0.11996
Thyroid	**0.05067**	**0.05067**	0.06083	0.08206	0.06728	0.06933
Heartstatlog	**0.16154**	**0.16343**	0.19129	0.16682	0.15647	0.17342
Ecoli	**0.15588**	**0.13980**	0.16796	0.16516	0.15877	0.16499
Glass	0.57879	0.57890	0.60825	0.56943	0.57092	0.57003
Jain	**0.01000**	**0.01000**	**0.02855**	0.05000	0.05000	0.05000
Vowel	**0.14382**	**0.14220**	**0.14974**	0.17170	0.17422	0.17374
Seeds	**0.04764**	**0.05504**	0.06730	0.04882	0.06160	0.06160
Sonar	0.18299	0.18421	0.21231	0.17410	0.20257	0.19467
Balance scale	0.21900	**0.17074**	0.25572	0.19357	0.19930	0.19926
Zoo	0.04597	0.04576	0.05720	0.03232	0.05771	0.06155

If the improved algorithm works better than or the same as the original BGOA, then we put the good result in bold font.

**Table 11:** The number of selected features.

Dataset	BGOA_S1	BGOA_S2	BGOA_V	BGOA	BPSO	BGWO
Air	33.66667	33.33333	**31.00000**	32.66667	36.00000	40.33333
Appendicitis	**5.66667**	**5.33333**	**1.00000**	6.00000	8.33333	6.66667
Austra	2.66667	4.33333	3.00000	2.33333	2.00000	2.00000
Balancescale	**2.33333**	6.00000	**0.33333**	4.00000	4.66667	5.66667
WDBC	**3.33333**	4.00000	**3.66667**	4.00000	4.00000	4.00000
Blood	12.00000	15.00000	16.66667	3.33333	12.33333	13.66667
Breast	0.00000	0.00000	0.00000	0.00000	0.00000	0.33333
Breast_gy	**3.33333**	5.33333	**4.66667**	5.33333	4.00000	4.00000
Bupa	**2.33333**	5.33333	5.66667	2.66667	4.00000	3.00000
Cleve	**3.33333**	4.00000	4.66667	4.00000	3.66667	4.00000
Cloud	**5.00000**	6.00000	6.33333	6.00000	6.33333	6.00000
Diabetes	**1.00000**	2.33333	3.33333	1.33333	1.66667	1.66667
Segmentation	4.00000	5.66667	4.00000	3.66667	2.33333	3.33333
Thyroid	**7.66667**	10.00000	8.33333	8.00000	8.00000	8.33333
Heartstatlog	2.66667	2.66667	2.33333	1.66667	1.66667	2.00000
Ecoli	5.66667	8.00000	8.33333	5.00000	6.66667	6.66667
Glass	5.00000	5.33333	5.33333	4.66667	4.33333	4.66667
Jain	**3.00000**	3.66667	5.33333	2.66667	3.66667	5.00000
Vowel	2.00000	2.00000	**1.66667**	2.00000	2.00000	2.00000
Seeds	**3.00000**	**3.00000**	**3.00000**	3.00000	3.00000	3.00000
Sonar	**2.00000**	2.66667	3.33333	2.00000	2.33333	2.33333
Balancescale	**13.66667**	**25.66667**	28.33333	32.66667	26.66667	31.66667
Zoo	**3.33333**	4.00000	**2.66667**	4.00000	4.00000	4.00000

If the improved algorithm works better than or the same as the original BGOA, then we put the good result in bold font.

**Table 12:** The accuracy of feature selection.

Dataset	BGOA_S1	BGOA_S2	BGOA_V	BGOA	BPSO	BGWO
Air	0.93077	**0.94687**	0.91923	0.95072	0.94277	0.95877
Appendicitis	0.95714	0.95714	0.94286	0.98571	0.96667	0.95714
Austra	0.86726	0.86488	0.85595	0.92024	0.86726	0.86786
Balancescale	**0.69162**	**0.68275**	**0.67306**	0.67012	0.67696	0.68247
WDBC	0.77947	0.85576	0.80789	0.87183	0.85904	0.86448
Blood	0.95801	0.96397	0.95741	0.97002	0.96239	0.96402
Breast	**0.76126**	**0.76118**	**0.76127**	0.75364	0.76122	0.76055
Breast_gy	0.75675	0.76254	0.74965	0.78263	0.76175	0.76368
Bupa	**0.79140**	**0.79465**	**0.77693**	0.76623	0.79491	0.78833
Cleve	0.66261	0.67639	0.63761	0.70561	0.68356	0.69033
Cloud	**0.82611**	0.82143	0.79865	0.82389	0.84206	0.83310
Diabetes	**0.99581**	0.99070	0.98466	0.99628	0.99351	0.99351
Segmentation	**0.75595**	0.74860	0.72870	0.76832	0.74115	0.74626
Thyroid	0.89127	**0.89810**	0.88667	0.89381	0.90286	0.89810
Heartstatlog	**0.95556**	**0.95556**	**0.94444**	0.93556	0.95111	0.95333
Ecoli	0.84123	0.84113	0.81326	0.84464	0.86228	0.84444
Glass	0.84976	**0.86649**	0.83804	0.86123	0.86546	0.86141
Jain	**0.41873**	**0.41937**	0.39159	0.41619	0.42048	0.42921
Vowel	**1.00000**	**1.00000**	0.97958	1.00000	1.00000	1.00000
Seeds	0.86483	0.86647	0.85885	0.87189	0.86924	0.86974
Sonar	**0.95476**	0.94825	**0.93683**	0.96365	0.95270	0.95270
Balancescale	**0.81746**	**0.81825**	**0.79032**	0.84540	0.81016	0.82286
Zoo	0.78721	0.83763	0.74843	0.84887	0.84285	0.84288

If the improved algorithm works better than or the same as the original BGOA, then we put the good result in bold font.

**Table 13:** The result of Wilcoxon rank-sum test.

Dataset	BGOA_S1	BGOA_S2	BGOA_V	BPSO	BGWO
Air	0.1746	0.4444	0.0079	0.0397	0.0079
Appendicitis	0.5397	0.4762	0.6508	0.8095	0.2460
Austra	0.7460	0.3095	0.8413	1.0000	0.4206
Balancescale	0.5714	1.0000	0.6905	0.0952	0.0317
WDBC	0.5873	1.0000	0.5714	0.4524	0.1746
Blood	1.0000	0.0079	0.1508	0.1429	0.4603
Breast	0.5714	0.4206	0.7302	0.0952	0.5873
Breast_gy	1.0000	0.1667	0.6905	0.1508	0.3095
Bupa	0.7460	0.2540	0.3095	0.0079	0.0079
Cleve	0.7460	0.1508	0.0317	0.0079	0.0079
Cloud	0.8571	0.3095	0.2381	0.8413	0.8413
Diabetes	0.1508	0.4206	1.0000	0.1508	0.1508
Segmentation	0.1349	0.0079	0.1508	0.0079	0.0317
Thyroid	0.0714	1.0000	0.1190	0.4762	0.1190
Heartstatlog	0.5476	0.6825	0.4365	0.0079	0.0159
Ecoli	0.0635	0.5079	0.0556	0.0079	0.0079
Glass	0.1349	0.8413	0.4206	0.0476	0.8016
Jain	1.0000	1.0000	1.0000	1.0000	1.0000
Vowel	0.5476	0.3968	1.0000	0.0079	0.0079
Seeds	0.9524	0.7063	0.2302	0.1190	0.0397
Sonar	0.1984	0.5476	0.0952	0.0556	0.0317
Balancescale	0.1508	0.3095	0.1032	0.0079	0.0079
Zoo	0.4921	1.0000	1.0000	0.0476	0.2063

**Table 14:** The result of Friedman test.

Dataset	Sum of Squares	Degree of Freedom	Mean Squares	*p*-Value
Air	5.2	2	2.6	0.2548
Appendicitis	0.4	2	0.2	0.0916
Austra	2.8	2	1.4	0.8557
Balancescale	2.8	2	1.4	0.0823
WDBC	0.7	2	0.35	0.0382
Blood	3.6	2	1.8	0.4060
Breast	1.9	2	0.95	0.1132
Breast_gy	0.4	2	0.2	0.8995
Bupa	3.6	2	1.8	0.0427
Cleve	0	2	0	0.1257
Cloud	0	2	0	0.1018
Diabetes	0.3	2	0.15	0.4830
Segmentation	4.9	2	2.45	0.0342
Thyroid	4.8	2	2.4	0.0513
Heartstatlog	0	2	0	0.6151
Ecoli	0.4	2	0.2	0.2311
Glass	1.2	2	0.6	0.0663
Jain	2.8	2	1.4	0.0174
Vowel	3.6	2	1.8	0.0427
Seeds	2.8	2	1.4	0.6151
Sonar	0.4	2	0.2	0.0427
Balancescale	3.6	2	1.8	0.1546
Zoo	1.9	2	0.95	0.0513

## Data Availability

Publicly available datasets were analyzed in this study. This data can be found here: http://archive.ics.uci.edu/ml/index.php, (accessed on 5 September 2021).
